# Evidence of rapid adaptive trait change to local salinity in the sperm of an invasive fish

**DOI:** 10.1111/eva.12859

**Published:** 2019-09-28

**Authors:** Leon Green, Jonathan N. Havenhand, Charlotta Kvarnemo

**Affiliations:** ^1^ Department of Biological and Environmental Sciences University of Gothenburg Gothenburg Sweden; ^2^ Linnaeus Centre for Marine Evolutionary Biology University of Gothenburg Gothenburg Sweden; ^3^ Department of Marine Sciences Tjärnö Marine Laboratory University of Gothenburg Gothenburg Sweden

**Keywords:** Baltic Sea, immigrant reproductive dysfunction, invasion biology, local adaptation, *Neogobius melanostomus*, salinity, spermatozoa

## Abstract

Invasive species may quickly colonize novel environments, which could be attributed to both phenotypic plasticity and an ability to locally adapt. Reproductive traits are expected to be under strong selection when the new environment limits reproductive success of the invading species. This may be especially important for external fertilizers, which release sperm and eggs into the new environment. Despite adult tolerance to high salinity, the invasive fish *Neogobius melanostomus* (round goby) is absent from fully marine regions of the Baltic Sea, raising the possibility that its distribution is limited by tolerance during earlier life stages. Here, we investigate the hypothesis that the spread of *N. melanostomus* is limited by sperm function in novel salinities. We sampled sperm from two invasion fronts with higher and lower salinities in the Baltic Sea and tested them across a range of salinity levels. We found that sperm velocity and percentage of motile sperm declined in salinity levels higher and lower than those currently experienced by the Baltic Sea populations, with different performance curves for the two fronts. Sperm velocity also peaked closer to the home salinity conditions in each respective invasion front, with older localities showing an increased fit to local conditions. By calculating how the sperm velocity has changed over generations, we show this phenotypic shift to be in the range of other fish species under strong selection, indicating ongoing local adaptation or epigenetic acclimation to their novel environment. These results show that while immigrant reproductive dysfunction appears to at least partly limit the distribution of invasive *N. melanostomus* in the Baltic Sea, local adaptation to novel environments could enable future spread beyond their current boundaries.

## 1 INTRODUCTION

For populations in novel environments, selection can contribute to adaptive trait change and with moderate to low gene flow, can result in local adaptations (Westley, Ward, & Fleming, 2013). Rapid evolution has been shown during the establishment of new populations in novel environments (Moran & Alexander, 2014; Reznick & Ghalambor, 2001; Westley, 2011). Different ecological processes can increase the rate of adaptation. First, phenotypic responses in individuals can occur as a direct reaction to adverse conditions (Purchase & Moreau, 2012). When these plastic responses are adaptive in the novel environment, they are naturally selected (West‐Eberhard, 2003). Secondly, traits associated with reproduction are by themselves commonly under strong selection (Hendry, 2016; Svensson et al., 2017). Gametes lack extensive supporting tissues (Lessells, Snook, & Hosken, 2009), which, together with the physiologically complex fertilization process, can make this stage into a bottleneck where individuals are further selected for the right environmental fit (Karr, Swanson, & Snook, 2009). To shield their gametes from an adverse environment, many organisms have developed internal fertilization. For aquatic animals with external fertilization, however, the gametes must be able to function in the surrounding water (Browne et al., 2015). Local microenvironments can have multiple impacts on reproduction, including DNA damage from salt stress or oxidative stress (Dowling & Simmons, 2009), sperm immotility (Beirão, Lewis, Wringe, & Purchase, 2018), incomplete egg binding (Herberg, Gert, Schleiffer, & Pauli, 2018), disrupted egg activation (Ginsburg, 1963) and egg penetration issues (Yanagimachi et al., 2017), and cause problems during DNA recombination (Iwamatsu & Ohta, 1978; Wai‐sum, Chen, & Chow, 2006). For invading species that are not adapted to these conditions, these mechanisms can cause ‘immigrant reproductive dysfunction’ (Svensson et al., 2017) and limit the establishment of a population in a novel environment.

Sperm movement in externally fertilizing fish is commonly triggered by changes in osmolarity or ion gradients at the release from the testes to the surrounding water (Browne et al., 2015; Islam & Akhter, 2012; Morisawa, 2008). As salinity is important also for the sperm velocity needed to compete during spawning, and therefore for fertilization success (Beirão et al., 2018; Gage et al., 2004; Gasparini, Simmons, Beveridge, & Evans, 2010; Purchase, 2018; Rudolfsen, Figenschou, Folstad, & Kleven, 2008), the ability to cope with a specific range of salinities is predicted to be under strong selection. This prediction is supported by observations that an organism's sperm function is commonly adapted to the salinity conditions of their reproductive habitat (Browne et al., 2015; Griffin et al., 1998; Morisawa, 2008; Svensson et al., 2017; Tiersch & Yang, 2012). This is not always true, however: recent studies of adaptation in sperm to environmental conditions have yielded surprising results. For example, the marine capelin, *Mallotus villosus*, evolved from a freshwater origin, has adapted so that its sperm are active immediately upon leaving the body. The strong negative effects of salinity on sperm velocity (Purchase, 2018) are overcome through high initial sperm velocity and short distance between egg and ejaculate (Beirão et al., 2018).

**Figure 1:**
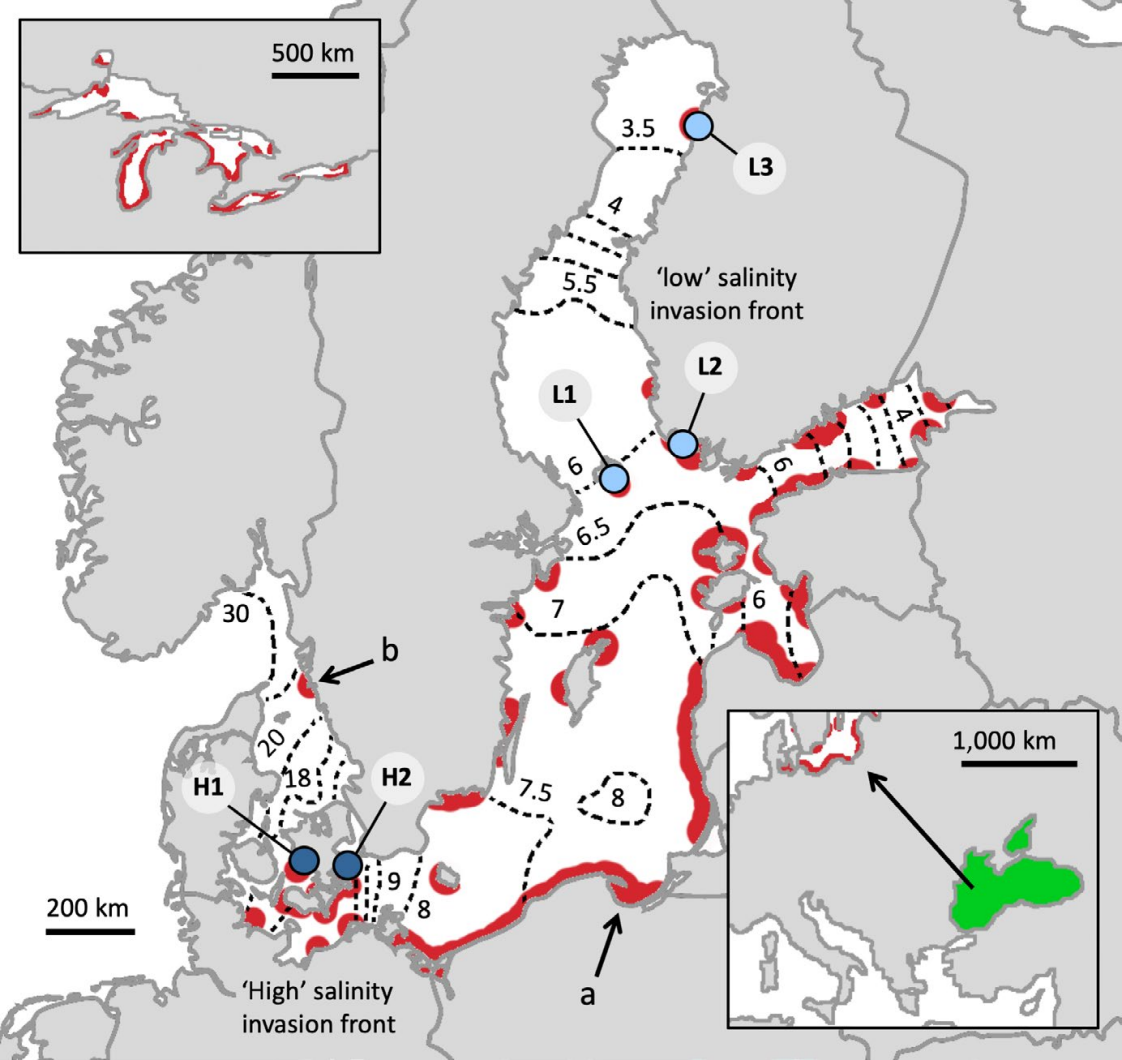
Map of sampled localities, *Neogobius melanostomus* range and the salinity gradient of the Baltic Sea region. Top left panel shows spread in the Great Lakes in North America, and bottom right panel shows ancestral occurrence (green colour) in the brackish Black and Azov Seas (from Kornis et al., 2012). The site of first introduction of *N. melanostomus* into the Baltic Sea is indicated by the letter 'a'. Dotted lines show the broad scale salinity gradient by 0.5 PSU per line (Leppäranta & Myrberg, 2009). Red areas show the most recently published range of *N. melanostomus* in the Baltic Sea as referenced by Kotta et al. (2016). Blue dots mark our sampling sites of the two invasion fronts, pale blue for lower salinity (L1, L2 and L3) and dark blue for higher salinity (H1 and H2), (see Section 2.1 for further details). Arrows and letter 'b' indicate sites of interest (see Section 4). Table S1 provides environmental data related to localities

In the semi‐enclosed Baltic Sea, there is a salinity gradient from high (30 PSU, practical salinity units) to low (<2 PSU) from the Kattegat in the south‐west (Figure 1) to the Bothnian Bay in the north (Leppäranta & Myrberg, 2009). This salinity gradient is a major factor influencing what species can reproduce in a region: the frequency of externally fertilizing marine species in the Baltic Sea decreases with declining salinity, a pattern that is not evident for internal fertilizers (Törnroos & Bonsdorff, 2012). This salinity gradient has also promoted divergence in gamete function. For example, the brown algae *Fucus vesiculosus* in the northern Baltic Sea shows clonal, rather than the usual sexual, reproduction (Tatarenkov et al., 2005). Among fish, egg buoyancy of *Gadhus morhua* (Atlantic/Baltic cod) (Nissling & Westin, 1997) and egg viability and sperm velocity of the flatfish *Platichthys flesus* (Nissling, Nyberg, & Petereit, 2017) have also adapted to the local spawning conditions (~20 and 14 PSU) experienced by these Baltic populations.

The Baltic Sea is relatively species poor, due to its young age and unique salinity conditions (Bonsdorff, 2006; Snoeijs‐Leijonmalm, Schubert, & Radziejewska, 2016; Zettler et al., 2014). As a consequence, the region has a greater availability of niches that may become occupied by introduced species, especially those species originating from other brackish environments (Paavola, Olenin, & Leppäkoski, 2005). Introduction vectors such as heavy shipping traffic together with continued anthropogenic activity has allowed an increasing number of novel species to establish and spread throughout the basin (Leppäkoski et al., 2002).

One of the most extreme cases of a species introduction to the Baltic Sea is that of the round goby, *Neogobius melanostomus* ‘Pallas 1811’, previously *Apollonia melanostoma* (Gobiidae, Osteichtys), *N. melanostomus* is a small (≤30 cm) benthic dwelling fish with cryptic colouration. It occupies a niche as a micro‐predator, feeding on crustaceans, worms and mussels (Kornis, Mercado‐Silva, & vander Zanden, 2012). The species is endemic to the coastal areas of the brackish Black, Caspian, Azov and Marmara Seas, along with many freshwater tributaries in this area. It is known to tolerate temperatures between 0°C and 30°C and salinities ranging from 0 to 40 PSU (Kornis et al., 2012; Vassilev, Apostolou, Velkov, Dobrev, & Zarev, 2012). Whether this ability to persist across a range of environmental conditions is due to tolerance, plasticity or adaptation is not known. *N. melanostomus* has external fertilization and shows exclusive paternal care (Meunier, Yavno, Ahmed, & Corkum, 2009). During reproduction, 3‐ to 4‐year‐old males compete for nesting sites, where they attract 2‐ to 3‐year‐old females, court them and guard the demersal eggs until hatching (Kornis et al., 2012). Males sequentially spawn with many females, and sperm competition can be high due to parasitic spawnings (Bleeker, De Jong, Van Kessel, Hinde, & Nagelkerke, 2017; Marentette, Fitzpatrick, Berger, & Balshine, 2009).

The introduction of *N. melanostomus* to the Baltic Sea occurred in 1990 to the Bay of Gdansk in Poland (marked 'a' in Figure 1), as a consequence of trade shipping routes to the Black Sea after the fall of the Soviet Union (Brown & Stepien, 2008). At present, *N. melanostomus* shows two distinct invasion fronts: one into lower salinity waters of the Bothnian Bay and the Bay of Finland; and one into higher salinity waters, currently reaching the Danish Straits (Figure 1) where it is expanding at 30 km/year (Azour et al., 2015). While long‐distance spread (typically between major harbours) is likely to be shipping‐associated, local spread is also affected by both larval drift and adult dispersal and migration (Azour et al., 2015; Brownscombe & Fox, 2012; Christoffersen, Svendsen, Behrens, Jepsen, van Deurs, 2019).

No established *N. melanostomus* populations have been observed in the North Sea, despite experimental work showing that some adults can tolerate the typical salinity conditions of this region  (Behrens, Van Deurs, & Christensen, 2017). The species' absence has been explained by high mortality of larvae in high salinity waters (Karsiotis, Pierce, Brown, & Stepien, 2012). Alternatively, the biotic resistance hypothesis (Elton, 1958) argues that a lack of niche availability and stronger inter‐specific competition in more complex communities could explain their absence in the North Sea, as biodiversity in this region increases with salinity (Bonsdorff, 2006). Since salinity is likely to have a strong effect on fertilization and embryo development, another possible barrier could be immigrant reproductive dysfunction (Svensson et al., 2017). There are currently indications that *N. melanostomus* could overcome these barriers through local adaptation. For example, studies have found that populations of *N. melanostomus* can rapidly differentiate genetically and over short geographical distances, pointing to the potential to adapt to local conditions (Björklund & Almqvist, 2010). Strong founder effects (low genetic diversity) have also been found in fringe populations during expansion, potentially a sign of strong selection when moving into novel habitats (Brown & Stepien, 2009). Furthermore, gobies as a family show an extreme diversity of species and preferred habitats, indicative of fast local adaptation and niche specialization (Near et al., 2013; Svensson et al., 2017; Taylor & Hellberg, 2005; Thacker, 2014; Yamada, Sugiyama, Tamaki, Kawakita, & Kato, 2009). Spatial sorting, which can aid rapid adaptation (Shine, Brown, & Phillips, 2011), is also supported by differences found between invasion fronts and established areas in the species (Brandner, Cerwenka, Schliewen, & Geist, 2013; Thorlacius, Hellström, & Brodin, 2015).

Given the potential for phenotypic change to enable or possibly even promote colonization of invasive species into new environments (Hudson, McCurry, Lundgren, McHenry, & Shine, 2016; Westley, 2011), and the wide‐reaching effects of *N. melanostomus* on the ecosystem level (Kornis et al., 2012; Ojaveer et al., 2015), there is a strong argument to investigate possible signs of local adaptation in sperm performance, and evidence of reproductive restraint to their current range of salinity conditions.

### 1.1 Aims and predictions

In this study, our aim was to assess (a) whether the sperm of *N. melanostomus* in the Baltic Sea function at salinities found outside their current geographical range and (b) whether sperm from the two invasion fronts show differences in salinity tolerance. We hypothesized that Baltic *N. melanostomus* is geographically limited by reduced sperm movement in fully marine salinities (≥30 PSU), since the species is absent from these areas along the North Sea coast. We also predicted that sperm velocity would be highest in test conditions similar to the local salinity experienced by each population, and that sperm velocity in their home salinity would increase with time since introduction, since they would have had longer time to adapt.

## 2 MATERIALS AND METHODS

### 2.1 Sampling and environmental data

Experiments were conducted within the permit nr 86‐2013 issued by the Ethical Committee for Animal Research in Gothenburg. During the spring of 2015, adult and juvenile *N. melanostomus* were caught at five different localities in the Baltic Sea (Figure 1), three from the ‘low’ salinity invasion front: L1, L2 and L3 (Mariehamn, Turku and Raahe, Finland, respectively) and two from the ‘high’ salinity invasion front: H1 and H2 (Karrebaeksminde and Kindvig, Denmark) using a combination of fyke nets, baited cages and angling. Since the spawning season is determined by temperature (MacInnis & Corkum, 2000) and estimated to start at 9°C during spring, sampling was conducted when the water temperature had reached 10°C in each respective locality. Environmental data (surface salinity and temperature; Table S1) were recorded at each locality at 30 cm depth on the first day of catch using a salinometer (HQ30d Portable Meter fitted with a IntelliCAL Conductivity Standard Probe, Hach, calibrated with 0.05% NaCl standard solution) for three individual measurements to calculate an average. The salinity during the dates when the fish were caught at each locality was: L1 = 5 PSU, L2 = 4 PSU, L3 = 2 PSU, H1 = 13 PSU and H2 = 10 PSU. Of the caught fish (Figure S1), reproductively mature males were kept and held in their catch‐salinity in aerated closed‐circuit systems at 10°C for a maximum of 3 days before sampling. From each of the localities, a number of males (n = 10 [L1], n = 5 [L2], n = 7 [L3], n = 6 [H1] and n = 6 [H2]) were sampled for sperm to test sperm motility (see below).

### 2.2 Sperm movement parameters

We exposed the sampled sperm to seven different salinities (1, 5, 10, 15, 20, 25, 30 PSU). Filtered salt water (FSW) for sperm testing was made by filtering both North Sea coastal sea water of 30 PSU and freshwater from a drinking reservoir through a 25 µm filter before adding the freshwater to the sea water to dilute it to the desired calculated salinities, controlled by measuring with a freshly calibrated salinometer (same as above). The salinity samples were kept frozen in between experiments to avoid bacterial growth and thawed to 10°C temperature before use.

Before sampling, each *N. melanostomus* male was sacrificed by two concussive blows to the head quickly followed by decapitation and destruction of the brain. For each fish, testes and accessory glands (sperm duct glands) were then excised within 1 min. One of the testes was selected haphazardly and transferred into a 1.5 ml microcentrifuge tube (Eppendorf). This testis was cut five times using microsurgery scissors (stainless steel, curved, sharp point, 4 inch; Sigma‐Aldrich Co); then, 750 μl Ca‐free Ringer's solution (Karila, Jensen, & Holmgren, 1993) was added and the sample was stirred using a Vortex (Vortex‐Genie 2, Scientific Industries) for 3 × 1 s. The method was developed from the protocol set in Svensson et al. (2017). From this sample, 25 μl of sperm solution was transferred to a tube containing 750 μl of FSW at one of the seven different salinities, chosen at random, to create a ‘stock solution’ for subsequent tests. After a stock solution of a salinity was made, it was stirred 3 × 1 s using a Vortex‐Genie 2 and temperature regulated by storage for ~1 min in a thermal bath at 10°C before being tested. After sperm recording (see below) in one salinity condition, a new stock solution was made using the same procedure until sperm had been tested in all salinities in the above‐mentioned range.

To record sperm velocity and motility, a sample was chosen at random and the following procedure was conducted for each of the seven original samples: 45 μl was taken and transferred to a 2% (w/v) albumin‐coated glass slide fitted with an O‐ring. This slide was then covered with an albumin‐coated cover slip, acting as a lid to form a suspended drop (Havenhand & Schlegel, 2009). This procedure was repeated to create six technical replicates per male and salinity treatment. Each drop was then filmed using a high speed camera (PixeLINK PL‐D725) fitted to an inverted microscope (Axio Vert.A1; Carl Zeiss AG) at 10× magnification and standard contrast and illumination, for 15 frames (30 frames/s, size 2,592*2,048 pixels, exposure time 10 ms, gain 0, gamma 0.1). Sperm movement parameters from the videos were extracted using a computer‐assisted sperm analysis (CASA) plugin (Wilson‐Leedy & Ingermann, 2007) for ImageJ (National Institutes of Health) using the parameters shown in Table S2, following standard procedures (Purchase & Earle, 2012).

### 2.3 Casa data cleaning and statistical testing

In total, 1,428 video recordings were analysed. Technical replicates were all inspected visually by path trajectories in ImageJ. Eighteen recordings were removed from analysis because they showed faults due to optical aberrations (from tissue particles in the sample), and 16 additional recordings were removed because they showed artificial movement during filming (due to currents in the micro‐well). Another 18 erratically distributed technical replicates found to have 0 sperm swimming were removed from analysis (unless this was true for all replicates from the same individual and treatment), as these were attributed to malfunctioning camera software. All remaining technical replicates (*n* = 1,376) were then pooled for each male and test salinity and averaged for all the measured CASA parameters (see Table S2 and Data S1). We focused our analysis on per cent motile sperm and velocity of the curvilinear path (VCL), including only sperm that moved more than 25 μm/s, allowing comparison with previous studies (Locatello, Pilastro, Deana, Zarpellon, & Rasotto, 2007; Locatello, Poli, & Rasotto, 2013; Marentette et al., 2009).

Data were analysed statistically using linear mixed effects modelling through the *lme4* package (Bates, Mächler, Bolker, & Walker, 2015) in R version 3.3.3 (R Core Team, 2013) with ‘treatment salinity’ and ‘invasion front’ as fixed factors and ‘individual’ and ‘locality’ as random factors. The response variables tested were ‘VCL’ and ‘per cent motile sperm’. Since assumptions of normality of residuals and homogeneity of variances were not met for the natural data, it was transformed and the response variables were independently modelled. The analysis from each respective model with the best model fit (natural‐log‐transformed VCL and untransformed sperm motility) is presented in the results and discussion. We used Welch's two‐sample *t* tests for unequal variances as post hoc tests to compare at which salinities the two expansion fronts differed significantly.

### 2.4 Calculations of phenotypic change over time

To assess the rate of change in sperm velocity in the Baltic *N. melanostomus*, *Haldanes* were calculated for each invasion front. A *Haldane* (*H*), expressed as one standard deviation per generation, is traditionally calculated in a population over time (Gingerich, 1993), but is also commonly calculated using synchronous systems such as invasive populations in similar conditions but of different age (Westley, 2011). Our study populations differ in time since establishment: L1—4 years, L2—10 years, L3—4 years, H1—4 years and H2—2 years (Figure 1). We focused on the two localities from each invasion front that experience the most similar salinity conditions, but differ in their invasion age: L1, L2 and H1, H2. We excluded L3 in this comparison as its abiotic conditions differ markedly from L1 and L2 (for example lower summer temperatures due to higher latitude in L3). In contrast, L1 experiences seasonal conditions similar to L2 (these two sites are at the same latitude and area of the Baltic Sea [HELCOM, helcom.fi, October 2015]), as do H1 and H2. We used an  averaged ‘regional home’ salinity condition based on sampled salinity from each site and HELCOM data (same as above) (low invasion front = 5 PSU, high invasion front = 12.5 PSU) to which the sperm velocity would be expected to be adapted. Sperm velocity at 12.5 PSU was calculated as velocity measured at 10 PSU + velocity at 15 PSU divided by 2, for each individual fish, and then averaged for each population. The average age per generation used in the below calculation was 3 years. This value accounts for females being able to reproduce from their second year (MacInnis & Corkum, 2000), and males from their third, although younger males may use parasitic spawnings (Marentette et al., 2009). Phenotypic change over time was calculated using the following equation developed by Gingerich (1993):

$$rate(H)=\frac{\left(\frac{\ln X_{2}}{S_{\ln\;X}}\right)-\left(\frac{\ln X_{1}}{S_{\ln\;X}}\right)}{t_{2}-t_{1}}$$

in which ln *X*_2_ and ln *X*_1_ are the natural logarithms of the sample means from locality 2 and 1, *S*_ln_ *_X_* is the pooled standard deviation from the two localities, and *t*_2_ − *t*_1_ is the difference in generations since the time of establishment for the respective locality.

## 3 RESULTS

### 3.1 Sperm velocity

**Figure 2:**
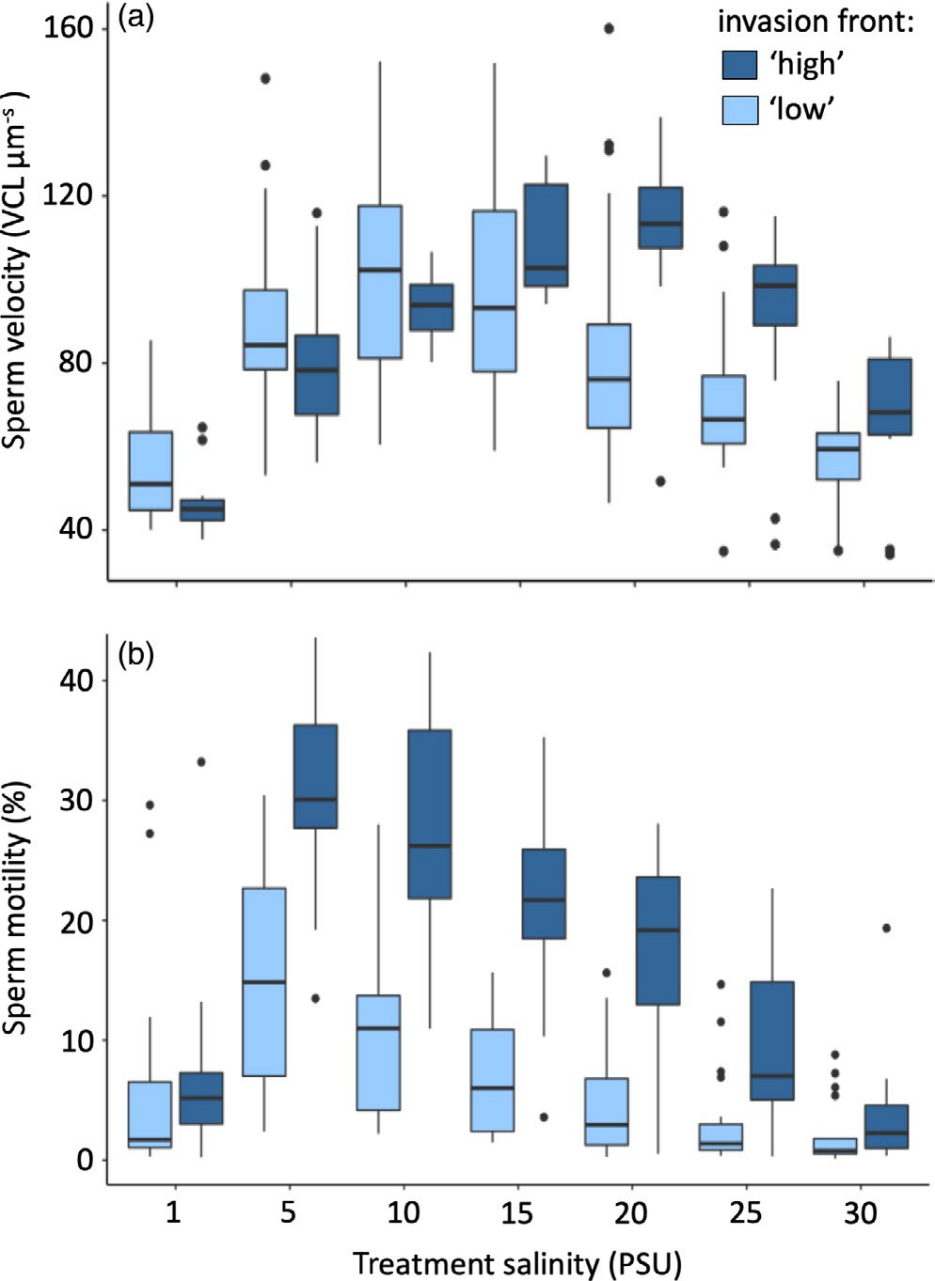
Sperm performance across the tested salinity conditions, with (a) sperm velocity and (b) sperm motility (per cent motile sperm) for fish sampled in the ‘high’ (dark blue) and ‘low’ (light blue) salinity invasion fronts. Boxplots show median, upper and lower quartile, and error bars show max and min, except outliers (dots) for fish from each invasion front, ignoring the level of site. Statistics are found in Table 1. Average and *SE* values for each locality in each treatment salinity are available in Table S3

We found a significant interaction between salinity treatment and invasion front on lnVCL (LMM, salinity (fixed) × invasion front (fixed): *t*_229.01, 7.63_ = −2.81, *p* = .005; Figure 2a, Table 1). The ‘high’ invasion front showed a trend towards higher velocity in 30 PSU (Welch's test, *t*_17.488_ = 1.76, *p* = .095), significantly higher velocity in 25 PSU (*t*_17.488_ = 2.38, *p* = .029) and 20 PSU (*t*_28.79_ = 3.14, *p* = .004) and significantly lower velocity at 1 PSU (*t*_31.557_ = −2.34, *p* = .026), compared with the ‘low’ invasion front. Other tested salinities (15, 10 and 5 PSU) did not differ (all *p* > .1).

**Table 1:** Results from linear mixed effects models of sperm velocity and motility. Salinity treatment and invasion front are included as fixed factors; individual and locality are included as random factors. Values in bold highlight significant effects.

Fixed effects	Estimate	*SE*	*df*	*t*	*p‐*Value
Response variable: velocity (lnVCL)					
Intercept	4.255	.094	7.630	45.184	**<.001**
Salinity	.009	.004	229.000	2.357	**.019**
Invasion front	.145	.120	7.120	1.214	.264
Salinity*invasion front	.013	.005	229.010	−2.811	**.005**

Response variable: percent motile sperm					
Intercept	49.083	6.163	4.940	7.964	**<.001**
Salinity	−.977	.192	228.940	−5.093	**<.001**
Invasion front	−25.241	7.877	4.740	−3.204	**.026**
Salinity*invasion front	.353	.240	228.940	1.470	0.143

Sperm were swimming at above 40 µm/s across all tested salinities and all tested localities, from 30 PSU (mean VCL ± *SE* = 61.02 μm/s ± 2.61) to 1 PSU (mean VCL ± *SE* = 52.31 μm/s ± 5.66) (Figure 2). Sperm velocity was lowest at 1 PSU in the ‘high’ invasion front (mean VCL ± *SE* = 52.50 μm/s ± 2.14). The peak in sperm velocity was at 20 PSU for the ‘high’ invasion front and at 10 PSU in the ‘low’ invasion front (Figure 2a and Table S3).

### 3.2 Sperm motility

The effects of salinity treatment and invasion front on sperm motility were significant, but there was no significant interaction (LMM, salinity [fixed]: *t*_228.94, 4.94_ = −5.093, *p* < .001; invasion front [fixed]: *t*_4.74, 4.94_ = −3.204, *p* = .026; salinity × invasion front: *t*_228.94, 4.94_ = 1.47, *p* = .14; Figure 2b; Table 1). The peak of sperm motility was at 5 PSU independent of locality or invasion front (Figure 2b), and motility in sperm from the ‘high’ invasion front was consistently higher in all but two salinities tested (Welch's *t* tests, *p* < .05 for 5–25 PSU).

### 3.3 Phenotypic change over time

**Figure 3:**
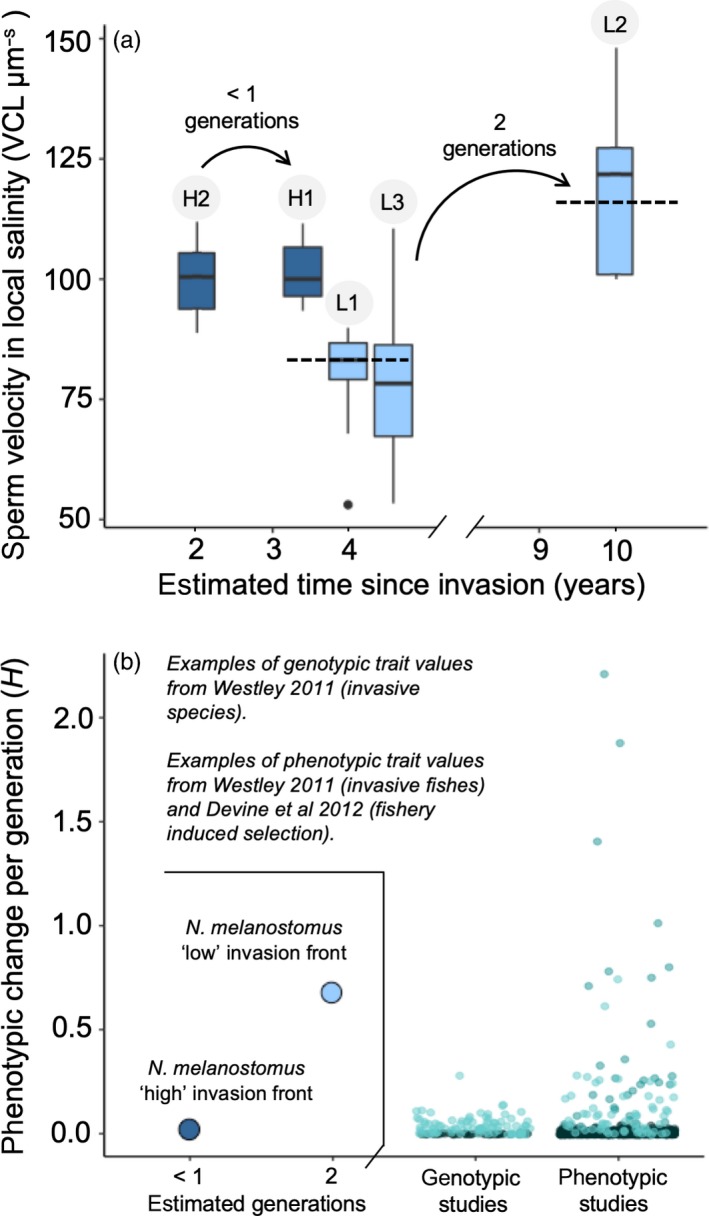
Phenotypic change over time. (a) Sperm velocity in regional home conditions (averaged from water samples and environmental data, 12.5 PSU for ‘high’ salinity and 5 PSU for ‘low’ salinity) compared with the estimated time since invasion. Dotted line shows average values for localities L1 and L2 used for calculations of Haldanes. The arrows highlight the difference in sperm velocity in populations established 2 and 6 years apart. (b) Left: A number of generations spent in the same salinity conditions affect phenotypic change over time. The ‘high’ salinity populations are estimated to be less than one generation apart in age, and little phenotypic change is expected or observed. The ‘low’ salinity populations are estimated to be at least two generations apart, and phenotypic change is both expected and observed. Calculation of Haldanes (*H* = change in standard deviations per generation) can be found in the material and methods section. Right: Haldanes for life history traits associated with reproductive success of other fish species under strong selection are included for effect size comparison. Data from genotypic studies on invasive species are from Westley (2011). Data of trait changes in phenotypic studies on fisheries from Devine et al. (2012) and invasive species are from Westley (2011). Data points are coloured as follows: light green: invasive species, black (bottom scatter): *Gambusia affinis* (separated due to overrepresentation in data) and dark green (phenotypic top scatter): traits under strong fishery selection

The difference in estimated age between our studied populations was 6 years for the low invasion front and 2 years for the high invasion front. In the low salinity invasion front, the L2 locality showed the highest sperm velocity (119.66 μm/s) in their regional home salinity (5 PSU). This locality is also reported as the oldest (10 years at time of sampling) (Figure 3a). Differences in sperm velocity between L1 and L2 give an *H* value of .675 standard deviations per generation. H1 and H2, which are estimated to be established 2 years apart, show little difference in sperm velocity at their regional home salinity (12.5 PSU, Figure 3a). Their *H* value was calculated to .01 standard deviations per generation. The difference in phenotypic change over time is shown in Figure 3b, together with examples of *H* of life history traits for other fishes under strong selection from fisheries or during species invasions (Devine, Wright, Pardoe, & Heino, 2012; Westley, 2011).

## 4 DISCUSSION

Our results show that salinity affects sperm velocity and sperm motility in *N. melanostomus*, but that sperm were still active at the high‐ and low‐end salinities that the Baltic *N. melanostomus* is expanding towards. Sperm from both invasion fronts maintained an average velocity above 40 µm/s in both 1 and 30 PSU treatments. This result thus rejects our prediction that intolerance to salinity in terms of sperm movement would offer a firm limit to the species' geographical spread. However, we show that fish from the two different invasion fronts in the Baltic Sea displayed different sperm velocities and sperm motility across the tested salinity range. The sperm of males sampled at the high salinity invasion front peaked in velocity at 15–20 PSU. This broadly matches (even exceeds) the salinity range from which the fish were caught (10–15 PSU, based on both regional salinity measurements and point samples during sampling). The ‘low’ salinity invasion front showed the highest velocity at 10 PSU, which indicates a shift towards the lower salinity range of these populations (2–5 PSU). These results thus support our second prediction, that the two invasion fronts would show signs of adaptive trait change to the local salinity.

### 4.1 Sperm show signs of adaptation to novel salinities

As a population enters a new adaptive landscape, trait performance curves can shift towards new optima due to selection (Hendry, 2016), such as the patterns seen in the sperm velocity differences between the ‘high’ and ‘low’ invasion fronts. We highlight this further by calculating the speed of phenotypic change per generation and compare this to other examples of fish populations under strong selection. These results show that the *Haldane* value for sperm velocity in the ‘low’ invasion front fits within the scope of previously reported traits under strong selection.

To assess the phenotypic change, it is important to consider the species' ancestral conditions. The ancestral Black Sea (Brown & Stepien, 2008) is brackish, with geographically variable salinity attributed to different upwelling currents and river mouth positions (Maximenko, Zatsepin, Panteleev, & Petyuh, 2012; Paavola et al., 2005). The range of salinity fluctuations *N. melanostomus* can tolerate is reported as 0–40 PSU based on occurrences in freshwater and in the hypersaline Aral Sea (Kornis et al., 2012), where the species was introduced and later disappeared (Khurshut, 2010). Genetic studies have shown that *N. melanostomus* that are found in the Baltic Sea originate from the Black Sea (Brown & Stepien, 2008) and the salinity conditions to which they are likely ancestrally adapted range from 11 to 18 PSU (Brown & Stepien, 2008), with 17–18 PSU being the most common (Maximenko et al., 2012; Paavola et al., 2005). Since the salinity conditions experienced by the high invasion front are closer to their ancestral environment, and selection on sperm is therefore likely to be more relaxed, low *Haldane* values would be expected for this area, which was what we observed (Figure 3b). The peak of sperm velocity seen in the current study at 15 and 20 PSU in the ‘high’ invasion front (Figure 2a) might reflect a pre‐adaptation (or a re‐adaptation, since the introduction into the Baltic Sea occurred at lower salinities) in their sperm performance to these conditions, and stabilizing selection in the local environment. Fish from Turku (L2, Figures 1 and 3), which were first reported in 2005, show the best fit to low salinity conditions. This locality also had the highest sperm velocity and sperm motility among the fish from the ‘low’ invasion front. Although we do not rule out additional effects of acclimation (occurring over an organism's lifetime), the increase in sperm velocity in lower salinity with time since establishment that we observed (Figure 3) indicates ongoing adaptation (occurring over generations). Since *N. melanostomus* females are able to reproduce in their second year (MacInnis & Corkum, 2000) and males in their third (sneaker tactics excluded), the fish in Turku have likely undergone adaptive selection over several generations within the 10 years since their establishment. In comparison, the other ‘low’ localities L1 and L3 were first observed in 2011 and have thus had considerably less time to respond to selection.

*Neogobius melanostomus* has been reported to show strong genetic differentiation over short time and short distances (Björklund & Almqvist, 2010; Brown & Stepien, 2009). Genomic traits causing rapid genetic change can also promote local adaptation to new salinities (Barth et al., 2017). Genes for adaptation to freshwater can be available in other populations and ‘sourced’ when selection promotes their persistence (Purchase & Moreau, 2012; Schluter & Conte, 2009). Given the potentially high connectivity within and between Baltic Sea localities and freshwater populations in mainland Europe due to extensive shipping traffic, there is substantial potential for genes to spread. Experiments show a broad salinity tolerance in the physiology of adults (Behrens et al., 2017; Hempel & Thiel, 2015; Karsiotis et al., 2012), which points to strong phenotypic plasticity. Other euryhaline fish have been reported to show phenotypic plasticity and acclimation of their sperm to different osmotic conditions during spermatogenesis (Kekäläinen et al., 2013; Taugbøl, Mazzarella, Cramer, & Laskemoen, 2017; Tiersch & Yang, 2012), but this has so far not been reported in gobies and should be investigated in future work. However, a strong plastic response to local salinity would have been expected to result in fish from different localities with similar salinity showing similar velocity. As shown in Figure 3, this was not the case.

The above evidence makes a strong case for adaptation through selection, but processes such as acclimation over a single generation (Taugbøl et al., 2017), or epigenetic inheritance over multiple generations (Rodríguez‐Romero, Jarrold, Massamba‐N’Siala, Spicer, & Calosi, 2016) cannot be ruled out. These processes can occur parallel to selection, but can also occur independently (Rohner, Roy, Schäfer, Blanckenhorn, & Berger, 2019).

An acclimation response (over a single generation) may potentially explain our results; however, this explanation requires that the sampled populations differ in ability to acclimate to the local salinity conditions. In the case of the low invasion front fish, the L2 population shows a strong match of velocity to the local environment and may be better at low salinity acclimation than L1, perhaps due to an ancestral adaptation to a variable environment (Brown & Stepien, 2008). Arguably, the older the age of the population, the more chances there would be of a plastic genotype to be transported to the region and get established. This scenario does however also include an aspect of adaptation, since the promotion of a plastic genotype over a nonplastic one infers selection during establishment.

A more likely but less well‐understood plasticity concept is an effect of trans‐generational plasticity, where gene‐expression patterns are inherited across generations, and potentially improved upon over multiple generations to better match the environment (Gibbin et al., 2017; Rodríguez‐Romero et al., 2016). Multi‐generational epigenesis is expected to show a similar pattern to local adaptation, and with our current knowledge, we cannot separate these two processes from each other in our study. The knowledge of how epigenesis interacts with local adaptation is also still in its infancy, and more research is needed to further our understanding of these potentially important processes.

Previous reports of *N. melanostomus* sperm velocity in freshwater averages around 100 μm/s (Marentette et al., 2009). Our highest measurements of *N. melanostomus* sperm velocity were in a similar range: velocity peaked at an average of 111 and 100 μm/s for the ‘high’ and ‘low’ invasion fronts, respectively. The high sperm velocity seen in *N. melanostomus* in the North American Great Lakes could be attributed to a freshwater‐adapted population, since the species is also found in freshwater tributaries to the Black Sea (Vassilev et al., 2012) in their ancestral region, and in many freshwater sites in continental Europe (Kornis et al., 2012). Future work on the populations' connectivity can address this question.

Overall sperm motility in our experiments was low. Most likely, this was caused by our sampling procedure, as cutting of the testes inevitably dilutes the sample of mature and motile sperm with immature spermatozoa incapable of movement. Since temperature has been reported as a strong factor influencing the onset of the species spawning period (Charlebois, Marsden, Wolfe, Jude, & Rudnicka, 1997), we sampled all the populations in the same temperature conditions. However, this experimental design could not simultaneously accommodate other environmental factors that could influence spawning readiness, such as seasonal and geographical light variation and adult energy status. Nevertheless, we found clear differences in sperm motility between the two invasion fronts (Figure 2b), with the ‘low’ front having fewer motile sperm overall. This may be an effect of less energy being available for reproduction, due to higher osmoregulatory costs, in lower salinities (Behrens et al., 2017). But the spawning behaviour of the fish might also affect this result, since the semi‐closed nest sites where fertilization occurs, as well as the prolonged spawning sessions (Meunier et al., 2009), limit the dilution effect for spawning males with few motile spermatozoa. Since the peak of sperm motility was at 5 PSU across all localities sampled, it is likely that selection on the number of active sperm is weak. This pattern seen in both fronts (Figure 2b) can reflect an ancestral adaptation not currently selected on in the new environment. For example, sperm numbers are often under strong selection during sperm competition (Pizzari & Parker, 2009), which in itself can be affected by a range of ecological variables (Monroe, Amundsen, Utne‐Palm, & Mobley, 2016). If population density is so low that there is no sperm competition, selection on sperm maturation rate (which may affect number of motile sperm or ejaculate volume) might not be as strong as natural selection on sperm velocity. As a consequence, local adaptation to salinity in terms of ejaculate volume could be a slow process and not yet visible in our sampled populations.

### 4.2 Adaptations and plasticity can increase the spread of *N. melanostomus*

*Neogobius melanostomus* has established itself in a range of freshwater habitats, both in its natural (Vassilev et al., 2012) and its invasive range (Kornis et al., 2012). An historical precedent for it spreading from brackish water into freshwater is known from its ancestral region (Vassilev et al., 2012). However, this also occurs on contemporary timescales with evidence of brackish fish invading tributaries that run into the Baltic Sea (Verliin et al., 2017). There is a high risk that more freshwater habitats in the region will become colonized by the species. We found that *N. melanostomus* sperm perform well in 5 PSU, and sperm of low invasion front males did better than high front males in 1 PSU. This ongoing phenotypic shift is expected to aid the species in colonizing freshwater from a brackish environment.

While *N. melanostomus* commonly colonizes the surrounding natural environment outside ports in the Baltic Sea (Kotta, Nurkse, Puntila, & Ojaveer, 2016), there are no reports of the species having colonized fully marine environments, despite occurring in adjacent brackish harbours. As adults can easily travel a few kilometres (Karsiotis et al., 2012), especially when densities get too high (Thorlacius et al., 2015), it is surprising that no marine populations have established themselves as of yet. However, if the ongoing adaptive trait change towards local salinity continues, successful reproduction of *N. melanostomus* is likely to occur in fully marine water in the future. We found sperm to be motile in 30 PSU for all sampled localities, with mean velocities ranging 55–74 μm/s. Despite these findings, their reproductive output may still be hampered in such environmental conditions. It remains to be tested whether sperm (as well as egg and larval development) are still functional at this top end of our tested salinity range, since motile sperm and adequate sperm velocity is not the only prerequisite for successful reproduction. Factors such as DNA damage and egg pathogens also affect the sensitive zygote development (Dowling & Simmons, 2009; Lehtonen & Kvarnemo, 2015) and hatching success (Purchase, 2018).

Since fish in the tested invasion fronts have not yet reached salinities beyond those experienced in their native range in the Black Sea region, it is difficult to estimate whether an adaptive trait change might dampen expansion speed. There are areas where *N. melanostomus* is present and where salinity varies by several PSU on a daily to monthly basis, such as Gothenburg harbour on the Swedish west coast (Figure 1, arrow b), and the Danish Straits (Leppäranta & Myrberg, 2009) where the high invasion front was sampled from. In these areas, the species may be selected for increased plasticity or tolerance due to the variable conditions. Increased acclimation ability can in turn enable a population to reach a fitness peak by prolonging its persistence in an adverse environment long enough for trans‐generational epigenesis and/or directional selection to have effect (Draghi & Whitlock, 2012; Price, Qvarnström, & Irwin, 2003; Scoville & Pfrender, 2010).

We acknowledge that acclimation and epigenetic effects are difficult to distinguish from adaptation (in the sense of gene‐frequency change), without studying the genomic signatures of selection. Future work on the study system will benefit greatly from these approaches, especially to fill in the gaps of knowledge in the interactions between the processes of plasticity and evolution (West‐Eberhard, 2003). Furthermore, behavioural traits (e.g., nest building) and effects of accessory gland content in the ejaculate are likely to have additional effects on the ability of *N. melanostomus* to spawn in different salinity conditions. Though this study examines the effect of salinity on reproduction, other ecological conditions also change with salinity, and abiotic factors such as temperature and pH are known to have strong effects on the reproductive biology of many species. These ecological conditions, whether related to food nutritional quality and food availability (Snoeijs‐Leijonmalm et al., 2016), interspecies competition (Kennedy et al. 2002) or niche availability (biotic resistance hypothesis) (Paavola et al., 2005) can all limit the extent of a species geographic boundaries. We urge researchers with expertise in these areas to apply their skills to the *N. melanostomus* study system to complete the picture of the species reproductive eco‐evolutionary dynamics.

## 5 CONCLUSIONS

We show that salinity affects sperm velocity and motility in *N. melanostomus,* that this effect varies across localities and, importantly, varies between the invasion fronts into ‘high’ and ‘low’ salinity waters in the Baltic Sea. This phenotypic change is likely to help *N. melanostomus* colonize and invade new areas. Since populations of freshwater *N. melanostomus* abound in continental Europe, and shipping traffic is high, the risk of a freshwater invasion from a brackish region would be unsurprising, despite our current result showing low sperm motility in salinity close to freshwater. Less well understood is the absence of an invasion of the North Sea coastline. Since *N. melanostomus* has not reached salinity conditions beyond those of their native waters of the Black Sea, it is hard to estimate how efficient the acclimation or adaptation of sperm traits can be in higher salinities. Considering the high invasiveness of the species, and the marked shift seen in the peak of sperm velocity between the invasion fronts in this study, it is crucial to further investigate their reproductive capacity throughout the colonization into both higher and lower salinities.

## CONFLICT OF INTEREST

The authors declare no competing interests.

## Data Availability

Data for this study are available at the Dryad Data repository https://doi.org/10.5061/dryad.77hm40j (Green, Havenhand, & Kvarnemo, 2019).
