# Supplementary material for: Evidence of rapid adaptive trait change to local salinity in the sperm of an invasive fish
Source: Evol Appl. 2019 Sep 28;13(3):533–44. doi: 10.1111/eva.12859 (PMC7045711; doi:10.1111/eva.12859)

Table S1. Environmental data for sampled localities ordered by date of catch. Black Sea is included for comparison. Salinities at the Baltic Sea locations are based on our own measurements, whereas salinity for the Black Sea is from Paavola et al*.* 2005 and Maximenko et al. 2012.

| **locality** | **name** | **invasion front** | **temperature**  **at catch** | **salinity**  **at catch** | **reported salinity of region** |
| --- | --- | --- | --- | --- | --- |
| H1 | Karrebaeksminde | High | 10°C | 13 PSU | 10 - 15 PSU |
| H2 | Kindvig | High | 10°C | 10 PSU | 10 - 15 PSU |
| L1 | Mariehamn | Low | 12°C | 5 PSU | 4 - 5 PSU |
| L2 | Turku | Low | 12°C | 4 PSU | 4 - 5 PSU |
| L3 | Raahe | Low | 10°C | 2 PSU | 3 PSU |
| - | Black Sea | - | - | - | 14 - 18 PSU |

| **first reported**  **observation** | **OSR**  **of catch** | **coordinates** | **date of catch** |  |
| --- | --- | --- | --- | --- |
| 2011 | 0.47 | 55°10'30.8"N 11°38'35.2"E | 25 - 26th of May 2015 |  |
| 2013 | 0.93 | 55°03'02.9"N 12°07'47.6"E | 27th of May 2015 |  |
| 2011 | 0.51 | 60°06'01.8"N 19°55'24.4"E | 4th of June 2015 |  |
| 2005 | 0.2 | 60°25'17.3"N 22°05'29.1"E | 13 - 14th of June 2015 |  |
| 2011 | 0.36 | 64°39'36.3"N 24°24'58.3"E | 11th of June 2015 |  |
| Native | - | - | - | |

Table S2. Settings used during tracking analysis for Sperm Tracker in CASA for ImageJ. Table shows order of settings as alphabetical characters, specific setting, units in brackets and responding value used during analysis. Software available from https://imagej.nih.gov/ij/plugins/casa.html during time of publishing.

| sperm tracker settings | value |
| --- | --- |
| a, Minimum sperm size (pixels): | 2 |
| b, Maximum sperm size (pixels): | 40.0 |
| c, Minimum track length (frames): | 5 |
| d, Maximum sperm velocity between frames (pixels): | 20 |
| e, Minimum VSL for motile (um/s): | 15 |
| f, Minimum VAP for motile (um/s): | 20.0 |
| g, Minimum VCL for motile (um/s): | 25.0 |
| h, Low VAP speed (um/s): | 25 |
| i, Maximum percentage of path with zero VAP: | 1.0 |
| j, Maximum percentage of path with low VAP: | 50 |
| k, Low VAP speed 2 (um/s): | 20.0 |
| l, Low VCL speed (um/s): | 25.0 |
| m, High WOB (percent VAP/VCL): | 80.0 |
| n, High LIN (percent VSL/VAP): | 80.0 |
| o, High WOB two (percent VAP/VCL): | 80.0 |
| p, High LIN two (percent VSL/VAP): | 80.0 |
| q, Frame Rate (frames per second): | 30.0 |
| r, Microns per 1000 pixels: | 480.0 |

Table S3. Sperm velocity (VCL) and sperm motility and for each invasion front and salinity treatment (tested PSU), sorted by invasion front and locality.

| **invasion front** | **locality** | **salinity treatment (PSU)** | **velocity (mean) (μm s-1)** | **velocity (S.E.)** | **motility (mean)** | **motility (S.E.)** |
| --- | --- | --- | --- | --- | --- | --- |
| HIGH | H1 | 1 | 49.09 | 4.53 | 19.51 | 9.70 |
|  |  | 5 | 85.20 | 6.55 | 56.01 | 8.95 |
|  |  | 10 | 96.18 | 2.03 | 49.21 | 9.15 |
|  |  | 15 | 106.86 | 5.71 | 33.40 | 6.55 |
|  |  | 20 | 105.73 | 11.66 | 25.18 | 7.08 |
|  |  | 25 | 81.74 | 13.65 | 12.64 | 4.52 |
|  |  | 30 | 60.34 | 8.57 | 5.00 | 1.90 |
|  | H2 | 1 | 44.67 | 1.32 | 10.54 | 4.06 |
|  |  | 5 | 76.66 | 8.61 | 68.79 | 5.13 |
|  |  | 10 | 90.04 | 4.32 | 62.95 | 6.64 |
|  |  | 15 | 110.16 | 5.75 | 53.98 | 6.02 |
|  |  | 20 | 115.86 | 5.16 | 45.07 | 5.84 |
|  |  | 25 | 97.03 | 4.43 | 26.34 | 6.90 |
|  |  | 30 | 74.20 | 4.22 | 11.41 | 5.87 |
| LOW | L1 | 1 | 52.73 | 4.16 | 8.72 | 2.53 |
|  |  | 5 | 79.65 | 3.58 | 36.77 | 4.81 |
|  |  | 10 | 88.70 | 5.76 | 25.29 | 3.82 |
|  |  | 15 | 87.84 | 6.31 | 17.90 | 3.47 |
|  |  | 20 | 72.63 | 4.66 | 8.16 | 2.61 |
|  |  | 25 | 58.91 | 3.45 | 5.52 | 2.38 |
|  |  | 30 | 54.95 | 4.47 | 3.46 | 1.83 |
|  | L2 | 1 | 55.18 | 8.12 | 30.51 | 11.69 |
|  |  | 5 | 119.66 | 8.98 | 40.24 | 8.07 |
|  |  | 10 | 127.88 | 7.69 | 31.26 | 7.96 |
|  |  | 15 | 115.37 | 17.45 | 16.82 | 4.60 |
|  |  | 20 | 101.79 | 22.68 | 15.86 | 6.43 |
|  |  | 25 | 83.93 | 8.06 | 11.03 | 5.20 |
|  |  | 30 | 60.76 | 2.31 | 6.54 | 2.92 |
|  | L3 | 1 | 59.88 | 4.15 | 1.98 | 0.32 |
|  |  | 5 | 78.48 | 7.14 | 13.88 | 4.25 |
|  |  | 10 | 96.63 | 11.79 | 9.64 | 3.07 |
|  |  | 15 | 96.74 | 7.81 | 7.64 | 2.18 |
|  |  | 20 | 84.45 | 8.28 | 6.62 | 1.58 |
|  |  | 25 | 75.59 | 7.15 | 3.47 | 0.75 |
|  |  | 30 | 57.84 | 5.84 | 3.14 | 1.61 |

Figure S1. Average total length of caught fish from different salinities in the ‘high’ and ‘low’ invasion fronts. Only positively sexed individuals (males and females) included. Error bars show S.E.. Abbreviations for localities can be found in table S1.


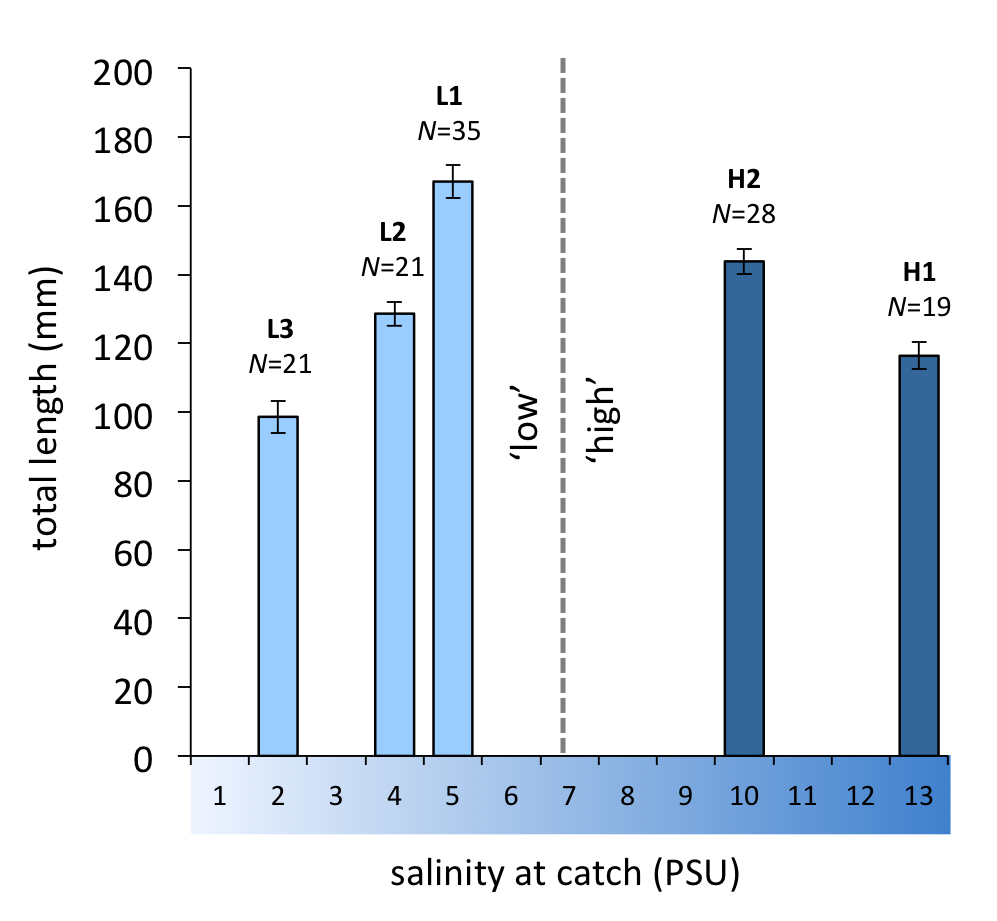

Supplement: Supplementary file 1 [file EVA-13-533-s001.docx]
